# Salt need needs investigation

**DOI:** 10.1017/S0007114520000173

**Published:** 2020-01-21

**Authors:** Micah Leshem

**Affiliations:** School of Psychological Sciences, The University of Haifa, Haifa 3498838, Israel

**Keywords:** Causes of salt intake, Salt appetite, Salt intake, Salt intake determinants, Salt intake research

## Abstract

Expensive and extensive studies on the epidemiology of excessive Na intake and its pathology have been conducted over four decades. The resultant consensus that dietary Na is toxic, as well as the contention that it is less so, ignores the root cause of the attractiveness of salted food. The extant hypotheses are that most Na is infiltrated into our bodies via heavily salted industrialised food without our knowledge and that mere exposure early in life determines lifelong intake. However, these hypotheses are poorly evidenced and are meagre explanations for the comparable salt intake of people worldwide despite their markedly different diets. The love of salt begins at birth for some, vacillates in infancy, climaxes during adolescent growth, settles into separate patterns for men and women in adulthood and, with age, fades for some and persists for others. Salt adds flavour to food. It sustains and protects humans in exertion, may modulate their mood and contributes to their ailments. It may have as yet unknown benefits that may promote its delectability, and it generates controversy. An understanding of the predilection for salt should allow a more evidence-based and effective reduction of the health risks associated with Na surfeit and deficiency. The purpose of this brief review is to show the need for research into the determinants of salt intake by summarising the little we know.

## Graphical abstract

**Figure:**
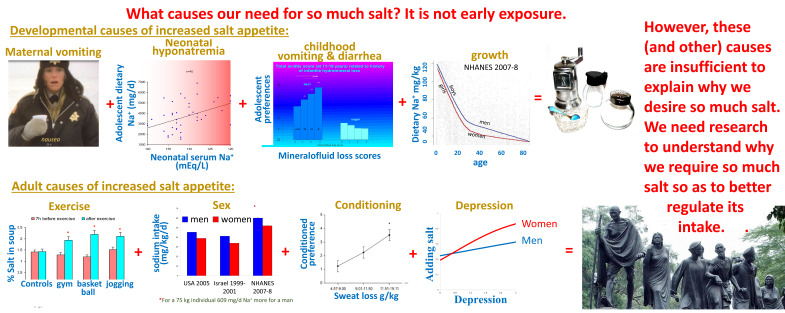

It took 85 % of the time life has existed on earth for animals to emerge onto dry land, and that occurred only when they could take with them the 0·9 % salty water that mimicked the primordial sea they relinquished^(1–3)^. Hence, for terrestrial animals, Na, the part constituent of common salt, is an indispensable, irreplaceable, life-supporting cation. In many animals, the means for acquiring and retaining it have evolved, respectively, Na appetite and kidneys. Indeed, Na demarcates the two forms of life by its motility, essential for animals but absent in plants.

For humans, salt may have initiated trade and urbanisation surrounding salt mines (European). Salt also serves religion and ceremony, and into the 20th century, its use for conserving food prevented starvation in both cold and hot climates^(4–6)^.

Today, worldwide, salt is consumed daily, repeatedly, totalling an amount that is in excess of that required to preserve life, which many hold to increase society’s disease burden, vascular and cancerous, significantly and cause three million deaths annually^(7)^. Obesity is estimated to cause four million deaths^(8)^, but a million of those may be due to salt intake^(9–11)^, so that while the two may be similarly deadly, the causes of obesity are researched incomparably more^(12,13)^. The reason for this is not clear, but it may be that obesity is prominently visible, whereas salt is allied to a silent killer, hypertension^(13)^.

The sole methods proposed to regulate salt intake are based on meagre evidence and their efficacy is dubious. It stands to reason that, if we knew the causes of salt intake, we could regulate it better^(14)^. The purpose of this brief review is to highlight the need for research into the determinants of salt intake by summarising the little we know.

## Critique of causes of salt intake and intervention

The determinants of our excessive salt appetite have been scarcely researched and, consequently, are scarcely understood^(14–18)^. Research has been primarily into the consequences of salt intake, primarily comprising large-scale studies, which have engendered the consensus that salt is toxic, along with a nuanced contention that it is less so^(7,9–11,18–27)^.

**Fig. 1.**
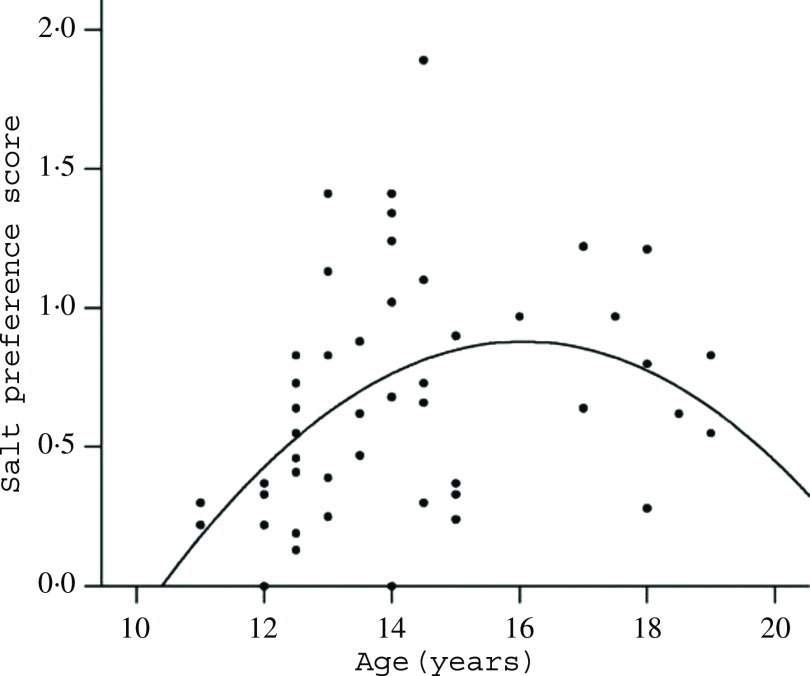
Salt preference in teens (*r* 0·64, *P* < 0·02). From Leshem^(16)^.

The extant hypotheses about the causes of excess salt intake are that mere exposure to salt early in life, together with Na infiltrated without our knowledge into our bodies via heavily salted processed food, determines our lifelong intake^(20,23,26,28,29)^. Yet, shoppers and diners may choose comparatively heavily salted food because salt enhances the flavours, rather than for its taste *per se*^(29,30)^, and salt intake is similar or greater where food is less industrialised^(7,24)^. Similarly, the evidence for early exposure as a determinant of later salt intake is poor, and many animal experiments have failed to confirm it^(15,16,31,32)^. The opposite is better evidenced: early Na restriction increases lifelong intake^(31–39)^. Moreover, growing children and adolescents ingest and prefer more salt than they were ever previously exposed to^(16,39,40)^ (Fig. 1). Hence, both extant hypotheses are meagre and unproven suppositions for a phenomenon as potent, pervasive and persistent as similar ingestion of salt across people with widely differing diets^(7,24,41)^.

Nevertheless, these hypotheses engender the prime methods advocated to regulate salt intake, advisory, admonitory and supervisory^(23)^. They are adopted widely, but selected instances have attained extraordinarily diverse results as measured over years (from an increase of 0·5 to a decrease of 4·8 g/d salt per individual^(23)^). However, long-term intervention studies and metanalyses included no comparison groups, provided no data on prior trends and rarely reported concomitant dietary and BMI changes which may determine Na intake. Moreover, they were confounded by sex, regional, and socio-economic differences, epochs of increases during intervention, different samples before and after intervention and changes in energy intake and diet^(23,41–46)^. Therefore, it is moot whether reduction is intervention-related any more than the parallel decrease in total energy intake, to which Na intake is inextricably linked^(45,46)^. Further, failures and contradictory data for the same countries in line with global increases or stability have also been reported^(14,18–28,42,47–51)^.

Excess salt intake has been related to many severe diseases, and yet it is uncertain how its use can be limited because too little salt may also contribute to ill health. People and communities differ so much that epidemiological studies, the mainstay of the Na–disease correlation, may swamp diversity, which may range from strong positive relationships to none or inverse relationships, even within the same study^(19,25–27,41,42,52–54)^. A J-shaped curve has been proposed to account best for the data^(19)^.

Wide acceptance of the infiltration and early exposure doctrines may divert us from efforts to understand the causes of high salt intake.

## Salt appetite and need

Salt appetite in animals, that is, its determinants and mechanisms, has been well studied. Studies have revealed that bodily Na deficit rapidly transforms the perception of the taste of even concentrated salt from repulsive to desirable. The transformation is mediated by extensive brain circuits, neurohormones and hormones^(2,55–57)^. Consistent with the benefit suggestion, salt consumption to alleviate a deficit frequently engenders a lifelong enhancement of salt appetite. It has been proposed that this is adaptive, prioritising salt by increasing its hedonic attraction, and hence salience, and storing Na sources in memory, all as increased protection to prevent hyponatraemic challenge, which has now become a proven hazard. The hazards have been suggested to be ecological (Na-scarce environments), constitutional or pathological (tendencies for, and individual causes of, dehydration and hyponatraemia)^(58–68)^.

While these physiological systems exist in humans too, the behaviour differs markedly. The remedial hedonic transformation is vestigial at best in humans and poorly evidenced. In fact, no reliable study of salt-deficient humans wanting salt spontaneously exists, and the studies that have been conducted failed to demonstrate it convincingly^(69–74)^, but see Leshem *et al.*^(75)^ and Wald & Leshem^(76)^. Indeed, in contradiction to studies in animals, even neonates^(59,77)^, studies in humans have found that they do not crave, seek or ingest salt when in need and can die from its lack in the body with salt at hand^(78)^.

In contrast to animals, whose salt consumption can be remedial, which is absent in humans, humans take pleasure from consuming salt with almost every food and meal. Daily it pleases all the inhabitants of the planet. Salt is invariably taken with food, which it enhances in many ways, increasing saltiness, suppressing bitterness, promoting taste where it is understated and imbuing it where absent, modifying texture and preserving, frequently when its own taste is covert^(15–17,56,79,80)^. Although this is consistent with the infiltration hypothesis as a cause of high salt intake, before acceding to it, recall the issue: why has our sense of taste evolved to respond in this way? It seems to be no coincidence because while the infinite variety of tastes and flavours is served by four taste receptors on the tongue, reinforced by olfaction, there is one more, unique among taste receptors in that it is dedicated solely to one ion – Na, the salt taste receptor (to which olfaction cannot contribute). There may even be one or two more, less specific, backup receptors^(81,82)^. No other nutrient, taste molecule or ion is awarded such specificity in humans or animals. These receptors, in addition to the taste of salt, also mediate some of its effects on other tastes via peripheral (oral) or brain-mediated neural activity^(81,83)^. Indeed, Na deficiency can impair other taste sensations^(71)^.

Thus, human salt appetite does not appear to be remedial as it is in animals, but it may be beneficial in other ways that enhance its taste to promote its intake.

## Acquisition of salt appetite

As already mentioned, it is generally believed that early exposure to salt in food determines lifelong intake, but the evidence is poor, and therefore, I shall detail what we do know.

The precocious rat pup brain has the salt appetite already at birth. By 12 d of age, the pup will lick salt if it requires it and, by weaning, it develops the ability to pinpoint Na among cations, possibly paralleling the process in the human fetus^(59)^.

**Fig. 2.**
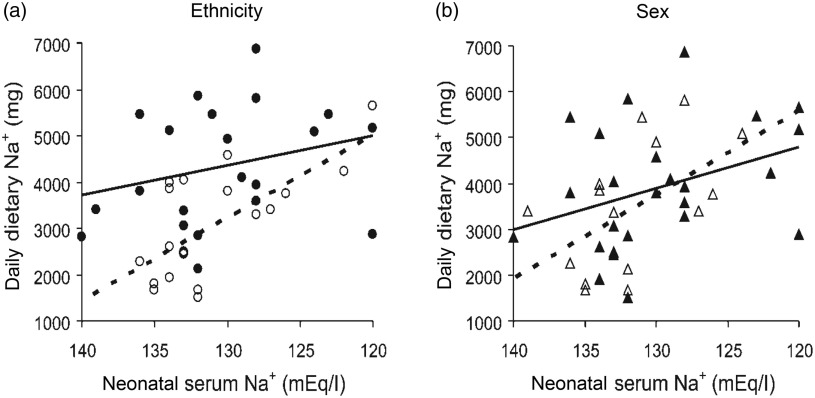
Correlations of neonatal serum and dietary sodium in children by (a) ethnicity and (b) sex. Black symbols and continuous lines, Arabs and boys, respectively; white symbols and dashes, Jews and girls, respectively. Correlations: Arabs, *r* 0·333 (NS, but without outlier, *r* 0·470*); Jews, *r* 0·520*; boys, *r* 0·549*; girls, *r* 0·400*. * *P* > 0·05. Data from forty-one children aged 10·5 (sem 0·2) years. From Shirazki *et al*.^(37)^, with permission.

Many preterm (about 10 % of babies) and some full-term babies are at risk of hyponatraemia and receive Na supplementation to ensure proper growth and neurological and cognitive development^(31,84–87)^. The severity of the obligatory neonatal dehydration and Na loss predicts the Na content of the diet we will compose in childhood and possibly beyond^(36–38,88)^ (Fig. 2).

**Fig. 3.**
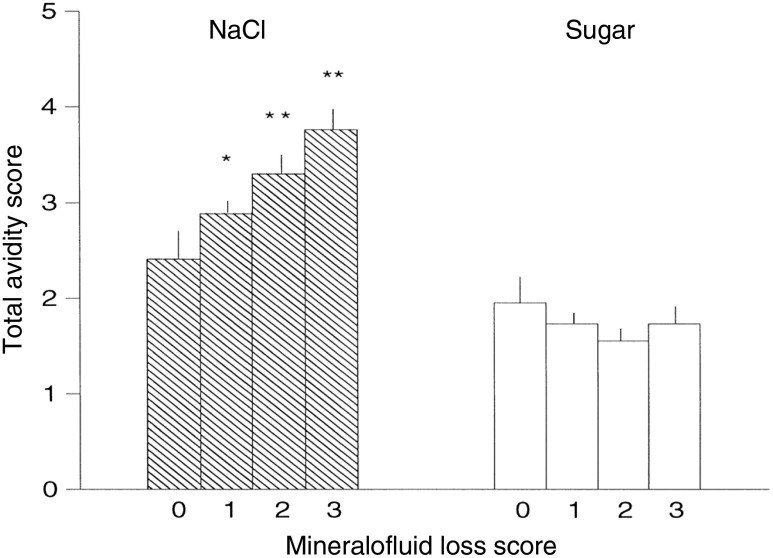
Relation of history of mineralofluid loss (maternal vomiting during pregnancy, infantile vomiting and diarrhoea) and avidity (sum of all test measures) for the taste of salt or sugar (the mean values with their standard errors for salt are higher than for sweet because they include scores for salting of food and dietary NaCl). 0 = no history of mineralofluid loss, 3 = highest incidence of mineralofluid loss. ** *P* > 0·01, different from 0. * *P* < 0·01, different from 3. The data are from fifty (8, 15, 18, 9 by mineralofluid loss score) girls and boys aged 14 (sem 2) years, and their mothers. From Leshem^(36)^ with permission. 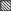, NaCl; 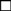, sugar.

Only some babies have a liking for salt^(34)^, in part dependent on birth weight, blood pressure^(37,89–91)^ and the severity of their mother’s morning sickness^(33–36)^. The severity of the mother’s morning sickness also has a long-term effect, increasing salt appetite in her offspring in their infancy, adolescence and adulthood^(33–36)^. Then, in infancy, childhood vomiting and diarrhoea contribute further to the perinatal influences increasing later salt appetite^(35–37,88)^ (Fig. 3). It is assumed that vomiting, whether maternal during pregnancy or in the child, and diarrhoea cause Na loss, thereby engaging the protective enhancement mentioned above.

As it develops, the human child increasingly has a predilection for salt, marginally related to early dietary experience^(32,92,93)^, but significantly related to neonatal hyponatraemia and to growth^(16,31,32,36–40,88,89)^. It is important to note that this increase in later salt appetite occurs with no experience of salt taste, a phenomenon established in rats^(2,58,65,66,94)^; in babies, the neonatal Na supplementation is administered intravenously which may not condition a salt preference^(95)^. In fact, it may be that the consequent increase in dietary Na of such children (aged 10–15 years) is unaccompanied by a preference for the taste of salt *per se*^(37)^ (but see Liem^(89)^), a known dissociation^(80,89,96)^. Finally, in the adolescent growth spurt, Na intake outstrips the intake of energy content, other macronutrients and electrolytes, together suggesting a unique developmental or maturational requirement^(39,40,84–86,97,98)^.

These observations, particularly that children’s salt intake is greater than adults’ salt intake, and boys’ salt intake greater than girls’ salt intake^(39)^, contradict the pervasive notion that ‘mere exposure’ to dietary salt, specifically early exposure, determines the subsequent attraction for salt and its intake. ‘Mere exposure’ is difficult to confirm in humans, but many studies in which rats were exposed to high dietary salt from gestation to adolescence have generally failed to reveal a systematic, Na-specific, relationship to long-term salt preference^(16,89)^.

Thus, in humans, its enhancement by early Na loss, restriction or deficiency is the most substantiated determinant of long-term salt appetite. The extent of the salt intake that is thus determined remains to be investigated, but morning sickness may affect 33 % of pregnancies^(33,99)^ and in a small study, increased salt intake in 50 % of adolescents was due to putative perinatal Na losses, a phenomenon consistent with other early metabolic programming^(31,32,36,85)^ (Figs. 2 and 3). Together, these could suggest a significant contribution to high salt intake in the population.

## Adult salt intake

However, in adults, Na loss, restriction or deficiency no longer enhances salt appetite^(100)^.

**Fig. 4.**
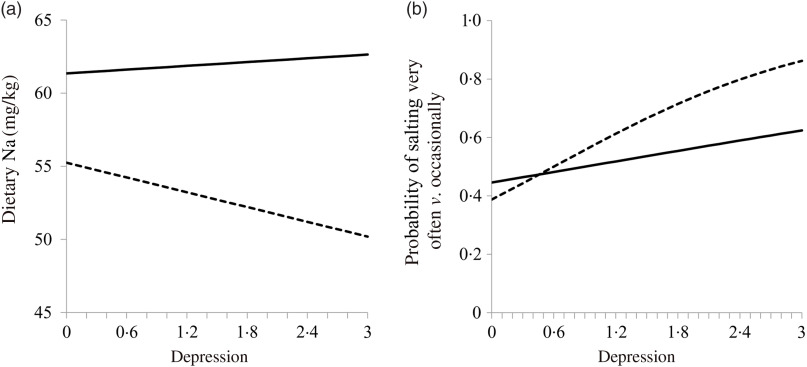
Relationship of weight-adjusted dietary sodium (a) and adding salt (b) to depression. Men, line; women, dashes. Both variables are adjusted for dietary energy. The relationships are significant for women. From Goldstein & Leshem^(39)^.

Adult salt intake is lower and settles into different patterns for men and women. Men take more salt per kg of body weight than women by about 20 %, possibly because men sweat more and have a greater lean mass^(16,76)^. Men’s higher intake also possibly protects them from depression because low dietary Na can contribute to depression, and women suffer more from depression^(39,101)^ (Fig. 4). In rats, low Na also indicates depression, and antidepressant treatment may reduce salt intake^(102,103)^. The relationship of salt intake and mood is examined briefly below.

## Salt and the elderly

Unlike other pleasures, sensations and tastes, such as thirst and hydration that wane with age, the taste for salt probably does not. Older people continue to relish their salt, and it may therefore be useful in maintaining their nourishment in age-related anorexia and hyponatraemia^(39,104–106)^. Older people are frequently hypertensive and hence routinely recommended to restrict Na intake, although some researchers have suggested the opposite advice might be given^(22,53)^. Cognitive impairment related to salt intake in the elderly has been studied, but the results are currently indeterminate^(105–109)^.

## Sodium deficiency

Dietary Na deficiency is rare, occurring in extreme cases of eating or drinking disorders^(110)^. Hyponatraemia, especially frequent among the institutionalised, hospitalised and the elderly, is associated with multiple pathologies, including of mood, and with mortality, and it is due to multiple fluid and electrolyte disorders^(111,112)^. Hyponatraemia is also frequent in physical exertion, due to Na loss in sweat or overhydration that leaches Na, particularly among less trained athletes. Nevertheless, athletes can be in mortal danger of hyponatraemic crisis because its diagnosis requires astute health workers^(78,111,112)^. These counsellors are necessary because unlike animals, humans seek salt to please their palate, but not to save their life^(78)^. Hence, many sports authorities recommend Na supplementation for safety, as well as to maintain athletic performance and accelerate recovery after it^(78,113–115)^. Such effects could condition a salt preference and contribute to its intake^(76)^.

Substantive findings suggest that low dietary Na contributes to CVD, whether in general or only in vulnerable individuals, as in the case of high Na intake, is not however known^(19,22,25,27,53,54)^. If Na intake alleviates the discomfort caused by these diseases, its taste may become preferred, and thus, its intake may be increased.

## Are there benefits supporting excess salt intake?

Na intake is essential to all bodily functions and to all organs, tissues, and cells, their membranes and contents, but current physiological knowledge indicates that a pinch a day suffices (about 1·3 g salt or 500 mg Na^+^), our intake far exceeds this amount. Evolutionary rationale suggests that prominent characteristics, such as perceiving the taste of salt as delectable, are readily explainable as adaptations, but it is not known how our excess salt intake may be beneficial^(15–17,39)^. Might there be benefits yet to be discovered driving this excessive intake^(39,116)^?

Benefits would tend to promote the inheritance of an increased salt appetite, whereas ailments such as hypertension, stroke and cancer would not tend to restrain it because evolutionary rationale biases for the inheritance of properties that are effective prior to reproductive age (the benefits), rather than after it (the ailments). Further, some of the maladaptive effects of Na may be adaptive in other circumstances^(62,116–118)^. Nevertheless, currently there is little evidence that salt appetite is inherited^(119–121)^.

Another suggested determinant is addiction. This implies that all 7·6 billion of humans are addicted to salt, despite the obvious fact that pure crystalline salt is not craved, ingested, injected or inhaled, even by people in putative withdrawal on a low-Na diet. This also dissociates animal studies that proposed that brain Na appetite substrates also serve other addictions^(2,122)^. Moreover, an addiction that is a norm might be a contradiction in terms, and dealing with it is rather daunting, considering our failure with other addictions, all of them together comparatively imperceptible^(123,124)^.

## Conditioning salt preference through exertion, mood, health and disease

Many physiological changes, modifying salt appetite or modified by it, may have little to do with maintaining Na homoeostasis. On the other hand, they may condition subsequent Na intake, if when Na is lost, salt intake alleviates some discomfort. This may underlie the increased acceptability of isotonic drinks in athletes and exercisers and in patients with Na-wasting diseases who discover salt to be prophylactic, and for the relief of hyponatraemia^(35,68,76,125)^.

In animal studies, salt was found to mitigate stress and facilitate social behaviour, which are useful effects, while low Na and its related hormones were found to indicate depression^(2,55,102,103,126)^. The human data are less consistent. Hostile male medical students seem to prefer salt^(127)^, and low dietary Na contributes to depression in Japanese men and marginally in American women, who may self-medicate with salt to improve their mood^(39,101)^. Other research has failed to relate the two or indicated cultural and dietary dependence, and yet other studies suggested salt may increase anxiety and even panic^(127–132)^.

Before or after exertion, many athletes drink Na-containing fluids and some swallow salt pills, and salt can aid recovery after exertion and condition a preference^(76,113–115)^. However, it is not known whether this generalises to the athlete’s dietary intake.

Desert dwellers relish salt, possibly to support hydration. They trade in salt and preserve their food in it, and it sustains their crucially important livestock and features prominently in their folklore^(118)^.

In some salt-wasting diseases, such as congenital adrenal hyperplasia, children frequently prefer salt to medication. It may ameliorate their affliction acutely and so may become favoured, whereas medication requires persistence and compliance, and even though its therapy is more comprehensive, long delayed effects condition poorly^(95)^.

Salt may mitigate pain^(132)^: dietary Na has an inconclusive direct relation to headache, is inversely related to migraine^(133)^ and may alleviate certain forms of fibromyalgia^(134,135)^. There is even a hypothesis that our high salt intake crucially protects us in the case of the many desiccating diseases^(62)^.

A long-standing issue of whether Na can be stored in the body has been resolved with the discovery of hypertonic Na in subcutaneous skin and muscle^(136)^. More importantly, immunity may be compromised by high salt intake, although some immune protection may be reduced with reduced salt^(137,138)^.

Such beneficial effects might condition a preference for the taste of salt, contributing to its intake^(35,76,123)^. Similar ideas have been considered for food intake, where palatability is central to the reinforcement hypothesis contributing to food intake and obesity^(2,125,139)^. Salt, of course, contributes greatly to palatability.

It is also possible that short-term negative effects condition reduced salt intake^(76)^, but their potential for regulation of salt intake has not been explored.

## Humans dislike salt and do not eat it

A very significant and frequently overlooked observation is that animals eat salt^(94,140)^, whereas humans do not^(16)^. Surprisingly, the delectability of salt for humans is unrelated to its taste. Very few people eat pure salt (an observation that militates against the addiction hypothesis).

Pure salt is inedible not merely because of it being concentrated (and activating aversive signalling taste receptors^(82,83)^), given that it is also aversive at low concentrations in water. This may be more than an issue of hedonism; it may be a physiological response because salt in solution is emetic^(141)^. Indeed, there are no salty drinks. Paradoxically, the same concentration (about 1 %) in an adulterated aqueous solution, such as tomato soup or beef broth, is relished^(16,56)^.

In contrast, animals lick rock salt, do not like salt in food (wherein its intake cannot be regulated^(142,143)^), prefer it in solution (wherein its intake can be regulated to the required 0·9 %^(143)^) and relish it most in the 0·9 % physiological concentration (like a saline drip). Hyponatraemic humans, however, require health workers to both diagnose their condition and administer Na^(68,78,111–114)^. Further, Na-deficient animals recognise Na in any mineral form^(67)^, whereas humans do not, taking only the single form, table salt (NaCl), suggesting that Na, the life-essential ion, is not the target cation taste as it is for animals^(16)^.

The comparison with the animal research is instructive because the animal behaviour, as outlined above, defines the behavioural requirements for the maintenance of Na homoeostasis, each of which humans abrogate, suggesting strongly that the humans’ love of salt in food does not stem from physiological Na requirement.

The causes must therefore be behavioural, with the caveat that there may well be specific requirements during early development and growth.

## Limitations

Confirmation, but particularly further research, of the determinants of salt intake is clearly required. Specifically, the significance of conditioning to excess Na intake is indeterminate. Research of this underrepresented science is limited at present, but resources for its encouragement should be found. The alternative notion that the excess salting of food has no palpable cause is not tenable.

## Conclusions

Throughout life, our love of salt peaks and dips. Salt flavours our food and promotes its consumption and thus possibly obesity; it sustains and protects us in physical exertion, may occasionally be remedial, contributes to our growth and ailments and generates controversy^(27)^.

Nonetheless, salt itself is inedible.

The attribution of this complexity to early dietary exposure and processed food is unsubstantiated, as well as inadequate.

The fundamental question persists of why we love the taste of salt.

An understanding of the predilection for salt taste should improve evidence-based intervention for effective reduction of the health risks associated with both Na surfeit and deficiency. For example, individual control of salt intake could benefit from counselling focused on children born to mothers who had high rates of nausea and vomiting during pregnancy, were hyponatraemic as neonates, or suffered Na losses in infancy, people with mood issues, and dissociating salt for exertion and diet for athletes. None of this is currently applied, and probably little known among those working to regulate Na intake. Note that salt need not be the direct cause of its associated effects, but can serve as the sensory marker for them as a ‘conditioned stimulus’ in conditioning theory.

However, most critical and promising are the determinants of salt intake, the discovery of which is surely awaiting novel and creative approaches in this crucial domain of human behaviour, nutrition and illness.

As astonishing science prepares to launch our first spaceship to Mars, it has yet to unravel the reasons for our daily 80 000-tonne sprinkle of salt^(41)^. Despite not knowing why we need so much salt, the ship will be supplied with it^(144)^.

## References

[1] Funder JW (2017) Aldosterone and mineralocorticoid receptors – physiology and pathophysiology. Int J Mol Sci 18, E1032. 28492512 10.3390/ijms18051032 PMC5454944

[2] Morris MJ, Na ES & Johnson AK (2008) Salt craving: the psychobiology of pathogenic sodium intake. Physiol Behav 94, 709–721. 18514747 10.1016/j.physbeh.2008.04.008 PMC2491403

[3] Rossier BC, Baker ME & Studer RA (2015) Epithelial sodium transport and its control by aldosterone: the story of our internal environment revisited. Physiol Rev 95, 297–340. 25540145 10.1152/physrev.00011.2014

[4] Kurlansky M (2002) Salt In A World History. New York: Walker & Company.

[5] Laszlo P (2001) Salt In Grain of Life [MB Mader, translator]. New York: Columbia University Press.

[6] Multhauf RP (1978) Neptune’s Gift: A History of Common Salt. Baltimore, MD: The Johns Hopkins University Press.

[7] GBD 2017 Diet Collaborators (2019) Health effects of dietary risks in 195 countries, 1990–2017: a systematic analysis for the Global Burden of Disease Study 2017. Lancet 393, 1958–1972. 30954305 10.1016/S0140-6736(19)30041-8 PMC6899507

[8] GBD 2015 Obesity Collaborators (2017) Health effects of overweight and obesity in 195 countries over 25 years. N Engl J Med 377, 13–27. 28604169 10.1056/NEJMoa1614362 PMC5477817

[9] Ma Y, He FJ & MacGregor GA (2015) High salt intake: independent risk factor for obesity? Hypertension 66, 843–849. 26238447 10.1161/HYPERTENSIONAHA.115.05948

[10] Zhang X, Wang J, Li J, et al. (2018) A positive association between dietary sodium intake and obesity and central obesity: results from the National Health and Nutrition Examination Survey 1999–2006. Nutr Res 55, 33–44. 29914626 10.1016/j.nutres.2018.04.008

[11] Zhou L, Stamler J, Chan Q, et al. (2019) Salt intake and prevalence of overweight/obesity in Japan, China, the United Kingdom, and the United States: the INTERMAP Study. Am J Clin Nutr 110, 34–40. 31111867 10.1093/ajcn/nqz067 PMC6599742

[12] Pubmed search terms: “Causes of obesity” 153,012 papers, “causes of salt intake” 5,243. “Health and obesity” 116,743, “health and salt” 30,547, “health and sodium 36,357”. (accessed April 2019).

[13] Shaldon S & Vienken J (2009) Salt, the neglected silent killer. Semin Dial 22, 264–266. 19573007 10.1111/j.1525-139X.2009.00606.x

[14] Burgermaster M, Rudel R & Seres D (2019) Interventions for dietary sodium restriction among patients with heart failure: A mismatch in the evidence and intervention design. Curr Dev Nutr 3, Suppl. 1, nzz028.OR22-05-19.

[15] Beauchamp GK (1987) The human preference for excess salt. Am Sci 75, 27–33.

[16] Leshem M (2009) Biobehavior of the human love of salt. Neurosci Biobehav Rev 33, 1–17. 18708089 10.1016/j.neubiorev.2008.07.007

[17] Mattes RD (1997) The taste for salt in humans. Am J Clin Nutr 65, Suppl. 2, 692S–697S. 9022567 10.1093/ajcn/65.2.692S

[18] Newson RS, Elmadfa I, Biro G, et al. (2013) Barriers for progress in salt reduction in the general population. An international study. Appetite 71, 22–31. 23891557 10.1016/j.appet.2013.07.003

[19] Graudal N, Hubeck-Graudal T, Jürgens G, et al. (2019) Dose-response relation between dietary sodium and blood pressure: a meta-regression analysis of 133 randomized controlled trials. Am J Clin Nutr 109, 1273–1278. 31051506 10.1093/ajcn/nqy384

[20] He FJ & MacGregor GA (2009) A comprehensive review on salt and health and current experience of worldwide salt reduction programmes. J Hum Hypertens 23, 363–384. 19110538 10.1038/jhh.2008.144

[21] He Y, Li Y, Yang X, et al. (2019) The dietary transition and its association with cardiometabolic mortality among Chinese adults, 1982–2012: a cross-sectional population-based study. Lancet Diabetes Endocrinol 7, 540–548. 31085143 10.1016/S2213-8587(19)30152-4 PMC7269053

[22] Mahtani KR, Heneghan C, Onakpoya I, et al. (2018) Reduced salt intake for heart failure: a systematic review. JAMA Intern Med 178, 1693–1700. 30398532 10.1001/jamainternmed.2018.4673

[23] Hyseni L, Elliot-Green A, Lloyd-Williams F, et al. (2017) Systematic review of dietary salt reduction policies: evidence for an effectiveness hierarchy? PLOS ONE 12, e0177535. 28542317 10.1371/journal.pone.0177535 PMC5436672

[24] McCarron DA (2014) What determines human sodium intake: policy or physiology? Adv Nutr 5, 578–84. 25469402 10.3945/an.114.006502 PMC4188239

[25] Messerli FH, Hofstetter L & Bangalore S (2018) Salt and heart disease: a second round of “bad science”? Lancet 392, 456–458. 30129446 10.1016/S0140-6736(18)31724-0

[26] Mozaffarian D, Fahimi S, Singh GM, et al. (2014) Global burden of diseases nutrition and chronic diseases expert group. Global sodium consumption and death from cardiovascular causes. N Engl J Med 371, 624–634. 25119608 10.1056/NEJMoa1304127

[27] Trinquart L, Johns DM & Galea S (2016) Why do we think we know what we know? A metaknowledge analysis of the salt controversy. Int J Epidemiol 45, 251–260. 26888870 10.1093/ije/dyv184

[28] Mancia G, Oparil S, Whelton PK, et al. (2017) The technical report on sodium intake and cardiovascular disease in low- and middle-income countries by the joint working group of the World Heart Federation, the European Society of Hypertension and the European Public Health Association. Eur Heart J 38, 712–719. 28110297 10.1093/eurheartj/ehw549

[29] Henney JE, Taylor CL & Boon CS (editors) (2010) Strategies to Reduce Sodium Intake in the United States. Washington, DC: National Academies Press. 21210559

[30] Liem DG & Russell CG (2019) The influence of taste liking on the consumption of nutrient rich and nutrient poor foods. Front Nutr 6, 174. 31803750 10.3389/fnut.2019.00174 PMC6872500

[31] Macchione AF, Caeiro XE, Godino A, et al. (2012) Availability of a rich source of sodium during the perinatal period programs the fluid balance restoration pattern in adult offspring. Physiol Behav 105, 1035–1044. 22133520 10.1016/j.physbeh.2011.11.015

[32] Mecawi AS, Macchione AF, Nuñez P, et al. (2015) Developmental programing of thirst and sodium appetite. Neurosci Biobehav Rev 51, 1–14. 25528684 10.1016/j.neubiorev.2014.12.012

[33] Crystal S & Bernstein IL (1995) Morning sickness, impact on offspring salt preference. Appetite 25, 231–240. 8746963 10.1006/appe.1995.0058

[34] Crystal SR & Bernstein IL (1998) Infant salt preference and mother’s morning sickness. Appetite 30, 297–307. 9632460 10.1006/appe.1997.0144

[35] Kochli A, Tenenbaum-Rakover Y & Leshem M (2005) Increased salt appetite in patients with congenital adrenal hyperplasia 21-hydroxylase deficiency. Am J Physiol Regul Integr Comp Physiol 288, R1673–R1681. 15650122 10.1152/ajpregu.00713.2004

[36] Leshem M (1998) Salt preference in adolescence is predicted by common prenatal and infantile mineralofluid loss. Physiol Behav 63, 699–704. 9523918 10.1016/s0031-9384(97)00525-8

[37] Shirazki A, Weintraub Z, Reich D, et al. (2007) Lowest neonatal serum sodium predicts sodium intake in low birth weight children. Am J Physiol Regul Integr Comp Physiol 292, R1683–R1689. 17170236 10.1152/ajpregu.00453.2006

[38] Stein LJ, Cowart BJ, Epstein AN, et al. (1996) Increased liking for salty foods in adolescents exposed during infancy to a chloride-deficient feeding formula. Appetite 27, 65–77. 8879420 10.1006/appe.1996.0034

[39] Goldstein P & Leshem M (2014) Dietary sodium, added salt, and serum sodium associations with growth and depression in the U.S. general population. Appetite 79, 83–90. 24747212 10.1016/j.appet.2014.04.008

[40] Quader ZS, Gillespie C, Sliwa SA, et al. (2017) Sodium intake among US school-aged children: National Health and Nutrition Examination Survey, 2011–2012. J Acad Nutr Diet 117, 39–47.e5 27818138 10.1016/j.jand.2016.09.010 PMC5458522

[41] Powles J, Fahimi S, Micha R, et al. (2013) Global, regional and national sodium intakes in 1990 and 2010: a systematic analysis of 24 h urinary sodium excretion and dietary surveys worldwide. BMJ Open 3, e003733. 10.1136/bmjopen-2013-003733 PMC3884590 24366578

[42] Public Health England (2016) National Diet and Nutrition Survey: Assessment of Dietary Sodium Adults (19 to 64 years) in England, 2014. https://assets.publishing.service.gov.uk/government/uploads/system/uploads/attachment_data/file/773836/Sodium_study_2014_England_Text_final.pdf (accessed June 2019).

[43] Barberio AM, Sumar N, Trieu K, et al. (2017) Population-level interventions in government jurisdictions for dietary sodium reduction: a cochrane review. Int J Epidemiol 46, 1551–1405. 28204481 10.1093/ije/dyw361 PMC5837542

[44] He FJ, Pombo-Rodrigues S & Macgregor GA (2014) Salt reduction in England from 2003 to 2011: its relationship to blood pressure, stroke and ischaemic heart disease mortality. BMJ Open 4, e004549. 10.1136/bmjopen-2013-004549 PMC3987732 24732242

[45] Public Health England (2019) National Diet and Nutrition Survey: Years 1 to 9 of the Rolling Programme (2008/2009–2016/2017): time trend and income analyses. https://assets.publishing.service.gov.uk/government/uploads/system/uploads/attachment_data/file/772434/NDNS_UK_Y1-9_report.pdf (accessed December 2019).

[46] O’Flaherty M, Buchan I & Capewell S (2013) Contributions of treatment and lifestyle to declining CVD mortality: why have CVD mortality rates declined so much since the 1960s? Heart 99, 159–162. 22962283 10.1136/heartjnl-2012-302300

[47] Bernstein AM & Willett WC (2010) Trends in 24-h urinary sodium excretion in the United States, 1957–2003: a systematic review. Am J Clin Nutr 92, 1172–1180. 20826631 10.3945/ajcn.2010.29367 PMC2954449

[48] Shankar B, Brambila-Macias J, Traill B, et al. (2013) An evaluation of the UK Food Standards Agency’s salt campaign. Health Econ 22, 243–250. 22223605 10.1002/hec.2772

[49] Okuda N, Stamler J, Brown IJ, et al. (2014) Individual efforts to reduce salt intake in China, Japan, UK, USA: what did people achieve? The INTERMAP Population Study for the INTERMAP Research Group. J Hypertens 32, 2385–2392. 25188367 10.1097/HJH.0000000000000341

[50] Pillay A, Trieu K, Santos JA, et al (2017) Assessment of a salt reduction intervention on adult population salt intake in Fiji. Nutrients 9, E1350. 29231897 10.3390/nu9121350 PMC5748800

[51] Public Health England (2018) Salt Targets 2017: Progress Report. https://assets.publishing.service.gov.uk/government/uploads/system/uploads/attachment_data/file/765571/Salt_targets_2017_progress_report.pdf (accessed December 2018).

[52] Intersalt Cooperative Research Group (1988) Intersalt: an international study of electrolyte excretion and blood pressure. Results for 24 hour urinary sodium and potassium excretion. BMJ 297, 319–328. 3416162 10.1136/bmj.297.6644.319 PMC1834069

[53] Mente A, O’Donnell M, Rangarajan S, et al. (2018) Urinary sodium excretion, blood pressure, cardiovascular disease, and mortality: a community-level prospective epidemiological cohort study. Lancet 392, 496–506. 30129465 10.1016/S0140-6736(18)31376-X

[54] Kong YW, Baqar S, Jerums G, et al. (2016) Sodium and its role in cardiovascular disease – the debate continues. Front Endocrinol 7, 164. 10.3389/fendo.2016.00164 PMC5179550 28066329

[55] Na ES, Morris MJ & Johnson AK (2012) Opioid mechanisms that mediate the palatability of and appetite for salt in sodium replete and deficient states. Physiol Behav 106, 164–170. 22326670 10.1016/j.physbeh.2012.01.019 PMC4433319

[56] Hayes JE, Sullivan BS & Duffy VB (2010) Explaining variability in sodium intake through oral sensory phenotype, salt sensation and liking. Physiol Behav 100, 369–380. 20380843 10.1016/j.physbeh.2010.03.017 PMC2874635

[57] Robinson MJ & Berridge KC (2013) Instant transformation of learned repulsion into motivational “wanting”. Curr Biol 23, 282–289. 23375893 10.1016/j.cub.2013.01.016 PMC3580026

[58] Leshem M, Kavushansky A, Devys JM, et al. (2004) Enhancement revisited: the effects of multiple depletions on sodium intake in rats vary with strain, substrain, and gender. Physiol Behav 82, 571–580. 15276824 10.1016/j.physbeh.2004.05.003

[59] Leshem M (1999) The ontogeny of salt hunger in the rat. Neurosci Biobehav Rev 23, 649–659. 10392657 10.1016/s0149-7634(98)00059-1

[60] Dietz DM, Curtis KS & Contreras RJ (2006) Taste, salience, and increased NaCl ingestion after repeated sodium depletions. Chem Senses 31, 33–41. 16306317 10.1093/chemse/bjj003

[61] Falk J (1966) Serial sodium depletions and NaCl solution intake. Physiol Behav 1, 75–77.

[62] Fessler DM (2003) An evolutionary explanation of the plasticity of salt preferences, prophylaxis against sudden dehydration. Med Hypotheses 613, 412–415. 10.1016/s0306-9877(03)00222-6 12944112

[63] Leshem M, Maroun M & Del Canho S (1996) Sodium depletion and maternal separation in the suckling rat increase its salt intake when adult. Physiol Behav 59, 199–204. 8848483 10.1016/0031-9384(95)02059-4

[64] Rowland NE & Fregly MJ (1988) Sodium appetite, species and strain differences and role of renin–angiotensin–aldosterone system. Appetite 11, 143–178. 3074734 10.1016/s0195-6663(88)80001-1

[65] Sakai RR, Fine WB & Epstein AN (1987) Salt appetite is enhanced by one prior episode of sodium depletion in the rat. Behav Neurosci 101, 724–731. 3675851 10.1037//0735-7044.101.5.724

[66] Sakai RR, Frankman SP & Fine WB (1989) Prior episodes of sodium depletion increase the need-free sodium intake of the rat. Behav Neurosci 103, 186–192. 2923672 10.1037//0735-7044.103.1.186

[67] Schulkin J (1991) Sodium Hunger: the Search for a Salty Taste. Cambridge: University Press.

[68] Montain SJ, Sawka MN & Wenger CB (2001) Hyponatremia associated with exercise: risk factors and pathogenesis. Exerc Sport Sci Rev 29, 113–117. 11474958 10.1097/00003677-200107000-00005

[69] Beauchamp GK, Bertino M & Burke D (1990) Experimental sodium depletion and salt taste in normal human volunteers. Am J Clin Nutr 51, 881–889. 2185626 10.1093/ajcn/51.5.881

[70] Henkin RI, Gill JR & Bartter FC (1963) Studies on taste thresholds in normal man and in patients with adrenal cortisol insufficiency, the role of adrenal cortical steroids and of serum concentration. J Clin Invest 42, 727–735. 16695903 10.1172/JCI104765 PMC289339

[71] McCance RA (1935–1936) Experimental sodium chloride deficiency in man. Proc R Soc B 119, 245–268. 10.1111/j.1753-4887.1990.tb02916.x 2406646

[72] Thorn GW, Dorrance SS & Day E (1942) Addison’s disease, evaluation of synthetic desoxycorticosterone acetate therapy in 158 patients. Ann Intern Med 16, 1053–1096.

[73] Wardener HE & Herxheimer A (1957) The effect of a high water intake on salt consumption, taste thresholds and salivary secretion in man. J Physiol 139, 53–63. 13481896 10.1113/jphysiol.1957.sp005874 PMC1358677

[74] Wilkins L & Richter CP (1940) A great craving for salt by a child with cortico-adrenal insufficiency. J Am Med Assoc 114, 866–868.

[75] Leshem M, Abutbul A & Eilon R (1999) Exercise increases the preference for salt in humans. Appetite 32, 251–260. 10097029 10.1006/appe.1999.0228

[76] Wald N & Leshem M (2003) Salt conditions a flavor preference or aversion after exercise depending on NaCl dose and sweat loss. Appetite 40, 277–284. 12798785 10.1016/s0195-6663(03)00013-8

[77] Wolf G (1969) Comments by Spector AC (2015) Innate mechanisms for regulation of sodium intake In Olfaction and Taste, pp. 548–553 [C Pfaffman, editor]. New York: Rockefeller University Press SSIB Ingestive Classics 2015. https://www.ssib.org/web/classic12.php (accessed May 2019).

[78] Moritz ML & Ayus JC (2008) Exercise associated hyponatremia: why are athletes still dying? Clin J Sport Med 18, 379–381. 18806542 10.1097/JSM.0b013e31818809ce

[79] Breslin PA & Beauchamp GK (1997) Salt enhances flavour by suppressing bitterness. Nature 387, 563. 10.1038/42388 9177340

[80] Lucas L, Riddell L, Liem G, et al. (2011) The influence of sodium on liking and consumption of salty food. J Food Sci 76, S72–S76. 21535718 10.1111/j.1750-3841.2010.01939.x

[81] Kure LC, Joseph PV, Feldman DE, et al. (2019) Brain imaging of taste perception in obesity: a eeview. Curr Nutr Rep 8, 108–119. 30945140 10.1007/s13668-019-0269-y PMC6486899

[82] Smith KR, Treesukosol Y, Paedae AB, et al. (2012) Contribution of the TRPV1 channel to salt taste quality in mice as assessed by conditioned taste aversion generalization and chorda tympani nerve responses. Am J Physiol Regul Integr Comp Physiol 303, R1195–R1205. 23054171 10.1152/ajpregu.00154.2012 PMC3533618

[83] Sandhu EC, Fernando ABP, Irvine EE, et al. (2018) Phasic stimulation of midbrain dopamine neuron activity reduces salt consumption. eNeuro 5, ENEURO.0064-18.2018. 10.1523/ENEURO.0064-18.2018 PMC5952649 29766048

[84] Al-Dahhan J, Jannoun L & Haycock GB (2002) Effect of salt supplementation of newborn premature infants on neurodevelopmental outcome at 10–13 years of age. Arch Dis Child Fetal Neonatal Ed 86, F120–F123. 11882555 10.1136/fn.86.2.F120 PMC1721384

[85] Alwasel SH, Barker DJ & Ashton N (2012) Prenatal programming of renal salt wasting resets postnatal salt appetite, which drives food intake in the rat. Clin Sci 122, 281–288. 21966935 10.1042/CS20110266

[86] Bischoff AR, Tomlinson C & Belik J (2016) Sodium intake requirements for preterm neonates: review and recommendations. J Pediatr Gastroenterol Nutr 63, e123–e129. 27276434 10.1097/MPG.0000000000001294

[87] Chan W, Chua MYK, Teo E, et al. (2017) Higher versus lower sodium intake for preterm infants. Cochrane Database Syst Rev, issue 4, CD012642. 10.1002/14651858.CD012642.pub2 PMC10569379 37824273

[88] Leshem M, Maroun M & Weintraub Z (1998) Neonatal diuretic therapy may not alter children’s preference for salt taste. Appetite 30, 53–64. 9500803 10.1006/appe.1997.0111

[89] Liem DG (2017) Infants’ and children’s salt taste perception and liking: a review. Nutrients 9, E1011. 28902163 10.3390/nu9091011 PMC5622771

[90] Zinner SH, McGarvey ST, Lipsitt LP, et al. (2002) Neonatal blood pressure and salt taste responsiveness. Hypertension 40, 280–285. 12215467 10.1161/01.hyp.0000029973.76439.ab

[91] Stein LJ, Cowart BJ & Beauchamp GK (2006) Salty taste acceptance by infants and young children is related to birth weight: longitudinal analysis of infants within the normal birth weight range. Eur J Clin Nutr 60, 272–279. 16306932 10.1038/sj.ejcn.1602312

[92] Sullivan SA & Birch LL (1990) Pass the sugar pass the salt, experience dictates preference. Dev Psychol 26, 546–551.

[93] Stein LJ, Cowart BJ & Beauchamp GK (2012) The development of salty taste acceptance is related to dietary experience in human infants: a prospective study. Am J Clin Nutr 95, 123–129. 22189260 10.3945/ajcn.111.014282 PMC3238456

[94] Leshem M, Langberg J & Epstein AN (1993) Salt appetite consequent on sodium depletion in the suckling rat pup. Dev Psychobiol 26, 97–114. 8467963 10.1002/dev.420260203

[95] Tordoff MG & Coldwell SE (2002) Some failures of intragastric NaCl infusions to support flavor preference learning. Physiol Behav 76, 511–519. 12126987 10.1016/s0031-9384(02)00724-2

[96] Drewnowski A, Henderson SA, Driscoll A, et al. (1996) Salt taste perceptions and preferences are unrelated to sodium consumption in healthy older adults. J Am Diet Assoc 96, 471–474. 8621872 10.1016/S0002-8223(96)00131-9

[97] Ayisi RK, Mbiti MJ, Musoke RN, et al. (1992) Sodium supplementation in very low birth weight infants fed on their own mothers milk I: effects on sodium homeostasis. East Afr Med J 69, 591–595. 1473517

[98] Bobowski N & Mennella JA (2019) Repeated exposure to low-sodium cereal affects acceptance but does not shift taste preferences or detection thresholds of children in a randomized clinical trial. J Nutr 149, 870–876. 31006818 10.1093/jn/nxz014 PMC6862934

[99] Chortatos A, Haugen M, Iversen PO, et al. (2013) Nausea and vomiting in pregnancy: associations with maternal gestational diet and lifestyle factors in the Norwegian Mother and Child Cohort Study. BJOG 120, 1642–1653. 23962347 10.1111/1471-0528.12406

[100] Leshem M (2009) The excess salt appetite of humans is not due to sodium loss in adulthood. Physiol Behav 98, 331–337. 19555703 10.1016/j.physbeh.2009.06.009

[101] Shimizu Y, Kadota K, Koyamatsu J, et al. (2015) Salt intake and mental distress among rural community-dwelling Japanese men. J Physiol Anthropol 34, 26. 26109460 10.1186/s40101-015-0064-4 PMC4480897

[102] De Gobbi JI, Omoto AC, Siqueira TF, et al. (2015) Antidepressant treatment decreases daily salt intake and prevents heart dysfunction following subchronic aortic regurgitation in rats. Physiol Behav 144, 124–128. 25747768 10.1016/j.physbeh.2015.02.037

[103] Leshem M (2011) Low dietary sodium is anxiogenic in rats. Physiol Behav 103, 453–458. 21453714 10.1016/j.physbeh.2011.03.025

[104] Hendi K & Leshem M (2014) Salt appetite in the elderly. Br J Nutr 112, 1621–1627. 25287294 10.1017/S0007114514002803

[105] Renneboog B, Musch W, Vandemergel X, et al. (2006) Mild chronic hyponatremia is associated with falls, unsteadiness, and attention deficits. Am J Med 119, 71.e1–71.e8. 10.1016/j.amjmed.2005.09.026 16431193

[106] Zallen EM, Hooks LB & O’Brien K (1990) Salt taste preferences and perceptions of elderly and young adults. J Am Diet Assoc 90, 947–950. 2195092

[107] Faraco G, Brea D, Garcia-Bonilla L, et al. (2018) Dietary salt promotes neurovascular and cognitive dysfunction through a gut-initiated TH17 response. Nat Neurosci 21, 240–249. 29335605 10.1038/s41593-017-0059-z PMC6207376

[108] Haring B, Wu C, Coker LH, et al. (2016) Hypertension, dietary sodium, and cognitive decline: results from the Women’s Health Initiative Memory Study. Am J Hypertens 29, 202–216. 26137952 10.1093/ajh/hpv081 PMC4723668

[109] Nowak KL, Fried L, Jovanovich A, et al. (2018) Dietary sodium/potassium intake does not affect cognitive function or brain imaging indices. Am J Nephrol 47, 57–65. 29393090 10.1159/000486580 PMC5815363

[110] Bhattarai N, Kafle P & Panda M (2010) Beer potomania: a case report. BMJ Case Rep 10, bcr1020092414. 10.1136/bcr.10.2009.2414 PMC3047485 22736559

[111] Hao J, Li Y, Zhang X, et al. (2017) The prevalence and mortality of hyponatremia is seriously underestimated in Chinese general medical patients: an observational retrospective study. BMC Nephrol 18, 328. 29089024 10.1186/s12882-017-0744-x PMC5664828

[112] Mohan S, Gu S, Parikh A, et al. (2013) Prevalence of hyponatremia and association with mortality: results from NHANES. Am J Med 126, 1127–1137.e1. 24262726 10.1016/j.amjmed.2013.07.021 PMC3933395

[113] Del Coso J, González-Millán C, Salinero JJ, et al. (2016) Effects of oral salt supplementation on physical performance during a half-ironman: a randomized controlled trial. Scand J Med Sci Sports 26, 156–164. 25683094 10.1111/sms.12427

[114] Evans GH, James LJ, Shirreffs SM, et al. (2017) Optimizing the restoration and maintenance of fluid balance after exercise-induced dehydration. J Appl Physiol (1985) 122, 945–951. 28126906 10.1152/japplphysiol.00745.2016

[115] Hew-Butler T, Loi V, Pani A, et al. (2017) Exercise-associated hyponatremia: 2017 update. Front Med 4, 21. 10.3389/fmed.2017.00021 PMC5334560 28316971

[116] Benton D, Cousins A & Young HA (2018) Why humans over-consume salt: it improves mood (P06–010) Curr Dev Nutr 2, nzy033.

[117] Mansley MK, Ivy JR & Bailey MA (2016) ISN Forefronts Symposium 2015: the evolution of hypertension-old genes, new concepts. Kidney Int Rep 1, 197–203. 27722209 10.1016/j.ekir.2016.08.003 PMC5044930

[118] Leshem M, Saadi A, Alem N, et al. (2008) Enhanced salt appetite, diet and drinking in traditional Bedouin women in the Negev. Appetite 50, 71–82. 17606311 10.1016/j.appet.2007.05.010

[119] Keskitalo K, Tuorila H, Spector TD, et al. (2008) The three-factor eating questionnaire, body mass index, and responses to sweet and salty fatty foods: a twin study of genetic and environmental associations. Am J Clin Nutr 88, 263–271. 18689360 10.1093/ajcn/88.2.263

[120] Kho M, Lee JE, Song YM, et al. (2013) Genetic and environmental influences on sodium intake determined by using half-day urine samples: the Healthy Twin Study. Am J Clin Nutr 9, 1410–1416. 10.3945/ajcn.113.067967 24088720

[121] Wise PM, Hansen JL, Reed DR, et al. (2007) Twin study of the heritability of recognition thresholds for sour and salty taste. Chem Senses 32, 749–754. 17623712 10.1093/chemse/bjm042 PMC2085364

[122] Liedtke WB, McKinley MJ, Walker LL, et al. (2011) Relation of addiction genes to hypothalamic gene changes subserving genesis and gratification of a classic instinct, sodium appetite. Proc Natl Acad Sci U S A 108, 12509–12514. 21746918 10.1073/pnas.1109199108 PMC3145743

[123] Cocores JA & Gold MS (2009) The salted food addiction hypothesis may explain overeating and the obesity epidemic. Med Hypotheses 73, 892–899. 19643550 10.1016/j.mehy.2009.06.049

[124] Tekol Y (2006) Salt addiction: a different kind of drug addiction. Med Hypotheses 67, 1233–1234. 16790320 10.1016/j.mehy.2006.04.041

[125] Yeomans MR, Blundell JE & Leshem M (2004) Palatability: response to nutritional need or need-free stimulation of appetite? Br J Nutr 92, Suppl. 1, S3–S14. 15384315 10.1079/bjn20041134

[126] Krause EG, de Kloet AD, Flak JN, et al. (2011) Hydration state controls stress responsiveness and social behavior. J Neurosci 31, 5470–5476. 21471383 10.1523/JNEUROSCI.6078-10.2011 PMC3086063

[127] Miller SB, Friese M, Dolgoy L, et al. (1998) Hostility, sodium consumption, and cardiovascular response to interpersonal stress. Psychosom Med 60, 71–77. 9492243 10.1097/00006842-199801000-00016

[128] Wang CJ, Yang TF, Wang GS, et al. (2018) Association between dietary patterns and depressive symptoms among middle-aged adults in China in 2016–2017. Psychiatry Res 260, 123–129. 29182923 10.1016/j.psychres.2017.11.052

[129] Serim DB, Ozbek A, Ormen M, et al. (2017) Do mothers with high sodium levels in their breast milk have high depression and anxiety scores? J Int Med Res 45, 843–848. 28351282 10.1177/0300060517700013 PMC5536667

[130] Peskind ER, Jensen CF, Pascualy M, et al. (1998) Sodium lactate and hypertonic sodium chloride induce equivalent panic incidence, panic symptoms, and hypernatremia in panic disorder. Biol Psychiatry 44, 1007–1016. 9821565 10.1016/s0006-3223(98)00053-5

[131] Thi Thu Nguyen T, Miyagi S, Tsujiguchi H, et al. (2019) Association between lower intake of minerals and depressive symptoms among elderly Japanese women but not men: findings from Shika Study. Nutrients 11, E389. 30781841 10.3390/nu11020389 PMC6412241

[132] Foo H & Mason P (2011) Ingestion analgesia occurs when a bad taste turns good. Behav Neurosci 125, 956–961. 21928874 10.1037/a0025542 PMC3226930

[133] Pogoda JM, Gross NB, Arakaki X, et al. (2016) Severe headache or migraine history is inversely correlated with dietary sodium intake: NHANES 1999–2004. Headache 56, 688–698. 27016121 10.1111/head.12792 PMC4836999

[134] De Lorenzo F, Hargreaves J & Kakkar VV (1997) Pathogenesis and management of delayed orthostatic hypotension in patients with chronic fatigue syndrome. Clin Auton Res 7, 185–190. 9292244 10.1007/BF02267980

[135] Graham KF (2011) Dietary salt restriction and chronic fatigue syndrome: a hypothesis. Med Hypotheses 77, 462–463. 21680102 10.1016/j.mehy.2011.05.028

[136] Titze J (2014) Sodium balance is not just a renal affair. Curr Opin Nephrol Hypertens 23, 101–105. 24401786 10.1097/01.mnh.0000441151.55320.c3 PMC4932095

[137] Jantsch J, Schatz V, Friedrich D, et al. (2015) Cutaneous Na^+^ storage strengthens the antimicrobial barrier function of the skin and boosts macrophage-driven host defense. Cell Metab 21, 493–501. 25738463 10.1016/j.cmet.2015.02.003 PMC4350016

[138] Rucker AJ, Rudemiller NP & Crowley SD (2018) Salt, hypertension, and immunity. Annu Rev Physiol 80, 283–307. 29144825 10.1146/annurev-physiol-021317-121134 PMC5811318

[139] Epstein LH & Leddy JJ (2006) Food reinforcement. Appetite 46, 22–25. 16257474 10.1016/j.appet.2005.04.006

[140] Bowell R, Warren A & Redmond I (1996) Formation of cave salts and utilization by elephants in the Mount Elgon region, Kenya. Geol Soc Spec Publ 113, 63–79.

[141] Casavant MJ & Fitch JA (2003) Fatal hypernatremia from saltwater used as an emetic. J Toxicol Clin Toxicol 41, 861–863. 14677797 10.1081/clt-120025352

[142] Beauchamp GK & Bertino M (1985) Rats (*Rattus norvegicus*) do not prefer salted solid food. J Comp Psychol 99, 240–247. 4006437

[143] Stricker EM & Verbalis JG (1990) Sodium appetite In Handbook of Behavioral Neurobiology, vol. 10, *Neurobiology of Food and Fluid Intake*, pp. 387–419 [EM Stricker, editor]. New York: Plenum Press.

[144] Lerchl K, Rakova N, Dahlmann A, et al. (2015) Agreement between 24-hour salt ingestion and sodium excretion in a controlled environment. Hypertension 66, 850–857. 26259596 10.1161/HYPERTENSIONAHA.115.05851 PMC4567387

